# Supplementary material for: Metabolic reprogramming by Zika virus provokes inflammation in human placenta
Source: Nat Commun. 2020 Jun 11;11:2967. doi: 10.1038/s41467-020-16754-z (PMC7290035; doi:10.1038/s41467-020-16754-z)
Supplement: Supplementary file 1 — Supplementary Information [file 41467_2020_16754_MOESM1_ESM.pdf]

Supplementary Information

Metabolic Reprogramming by Zika Virus Provokes Inflammation in Human Placenta

Chen et al.

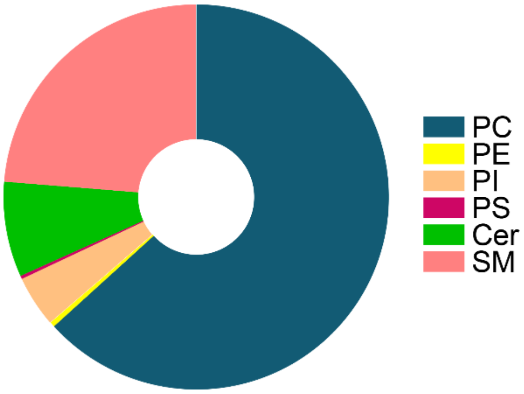

Supplementary Figure 1. Phospholipid Composition of First Trimester Human Placentas. Phosphatidylcholine (PC), Phosphatidylethanolamine (PE), Phosphatidylinositol (PI), Phosphatidylserine (PS), Ceramide (Cer) and Sphingomyelin (SM) are quantified by LC-MS/MS from 5 independent donors. All data are normalized to the protein amount and are presented as the percentage of total phospholipid amount. Source data are provided as a Source Data file.

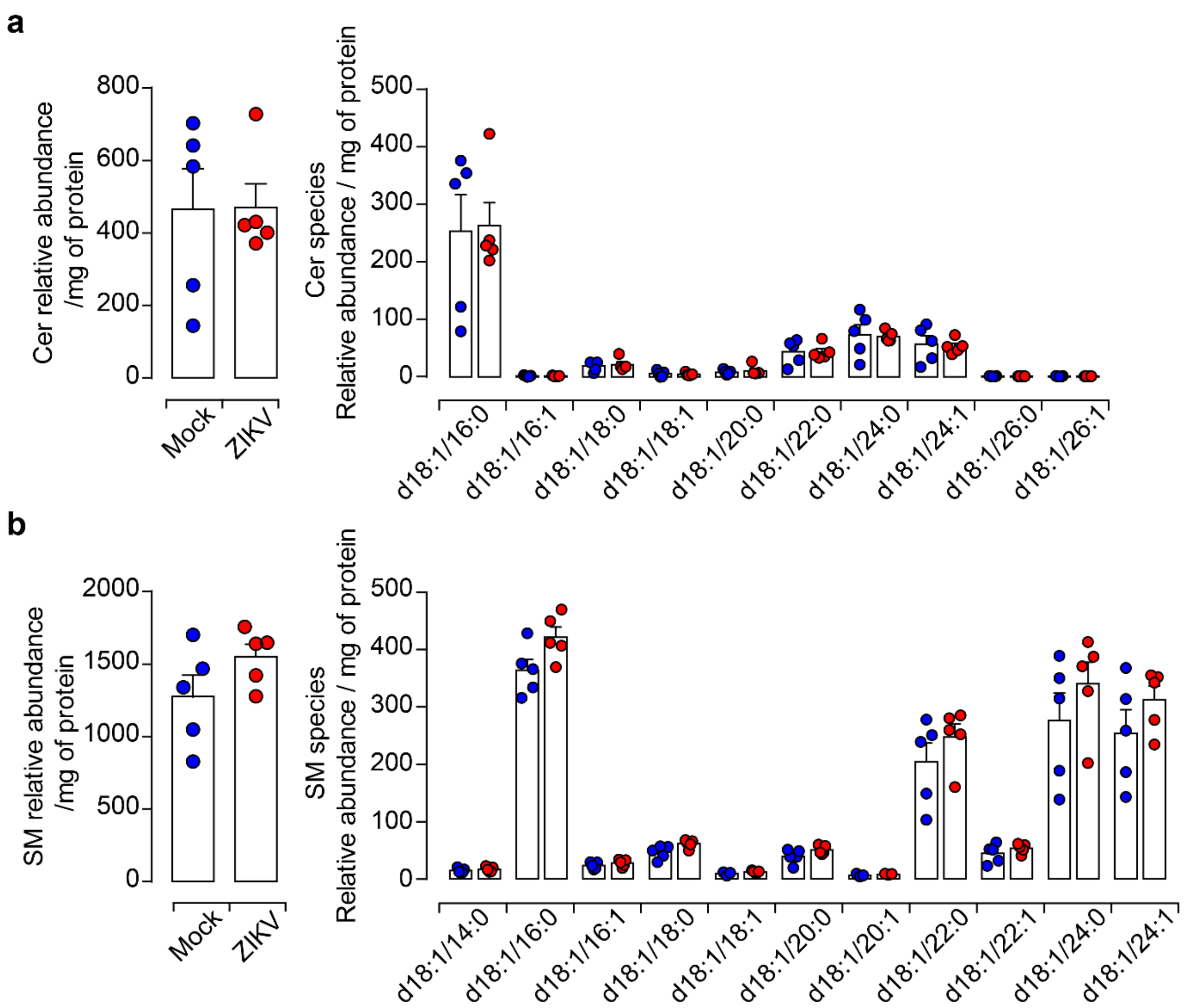

Supplementary Figure 2. Sphingolipid Profile of Mock and ZIKV Infected Placentas.

a, b Ceramide (Cer) and Sphingomyelin (SM) are quantified 5dpi from Mock- and ZIKV-infected placenta by LC-MS/MS. Ten lipid species from Cer (**a**) and Eleven lipid species from SM (**b**) are determined. No significant difference is observed between mock- and ZIKV-infected placentas. All data are normalized to the protein amount and are presented as mean values ± SEM from 5 independent donors. Source data are provided as a Source Data file.

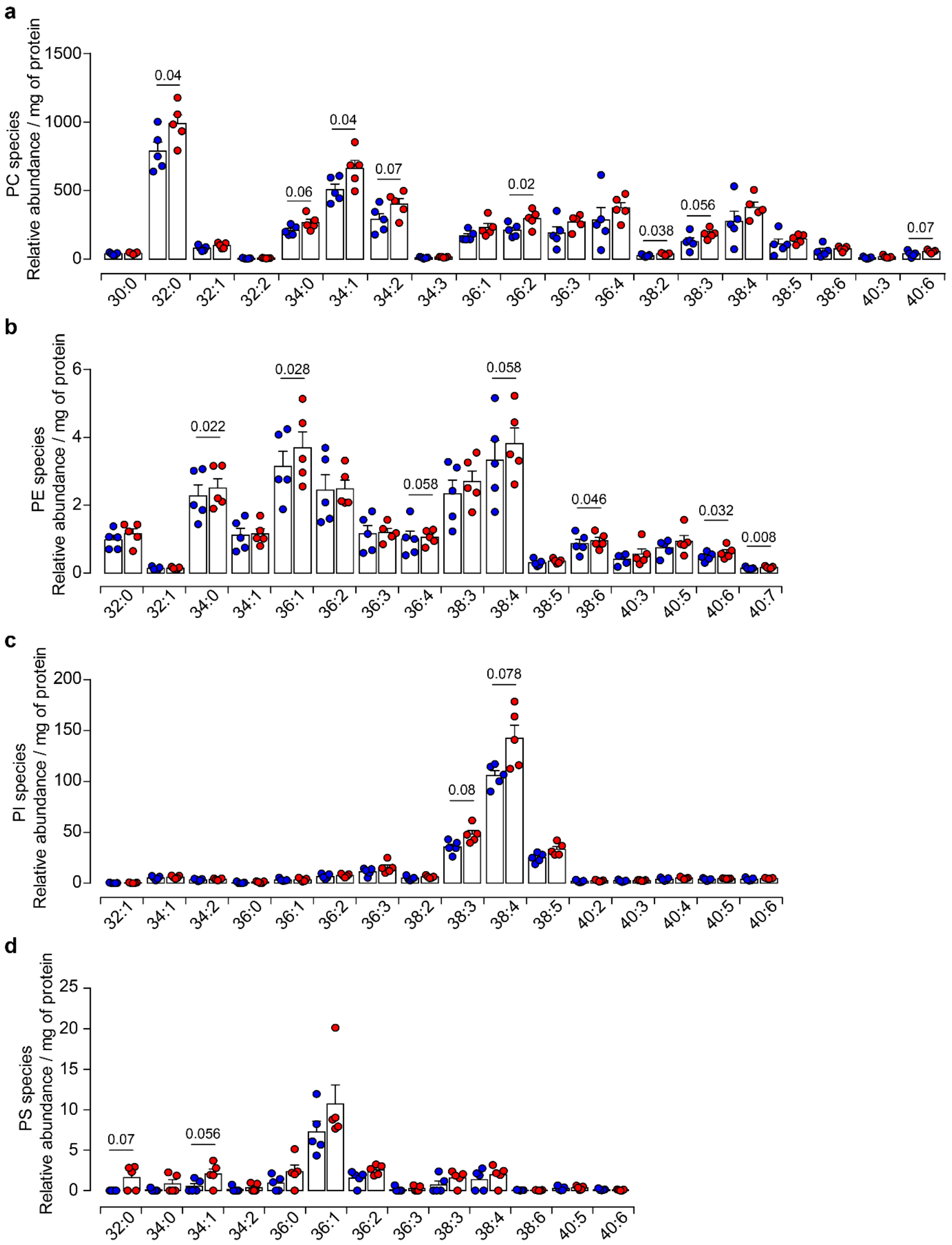

Supplementary Figure 3. Phospholipid Profiles of Mock and ZIKV Infected Placentas.

64 phospholipid subspecies quantified by LC-MS/MS. **a** Phosphatidylcholine (PC). **b** Phosphatidylethanolamine (PE). **c** Phosphatidylinositol (PI). **d** Phosphatidylserine (PS). All data are normalized to the amount of proteins and presented as mean values ± SEM from 5 independent donors. Significant difference between mock- and ZIKV-infected samples are calculated. *P* values are computed using the paired two-tailed Student's t test. Source data are provided as a Source Data file.

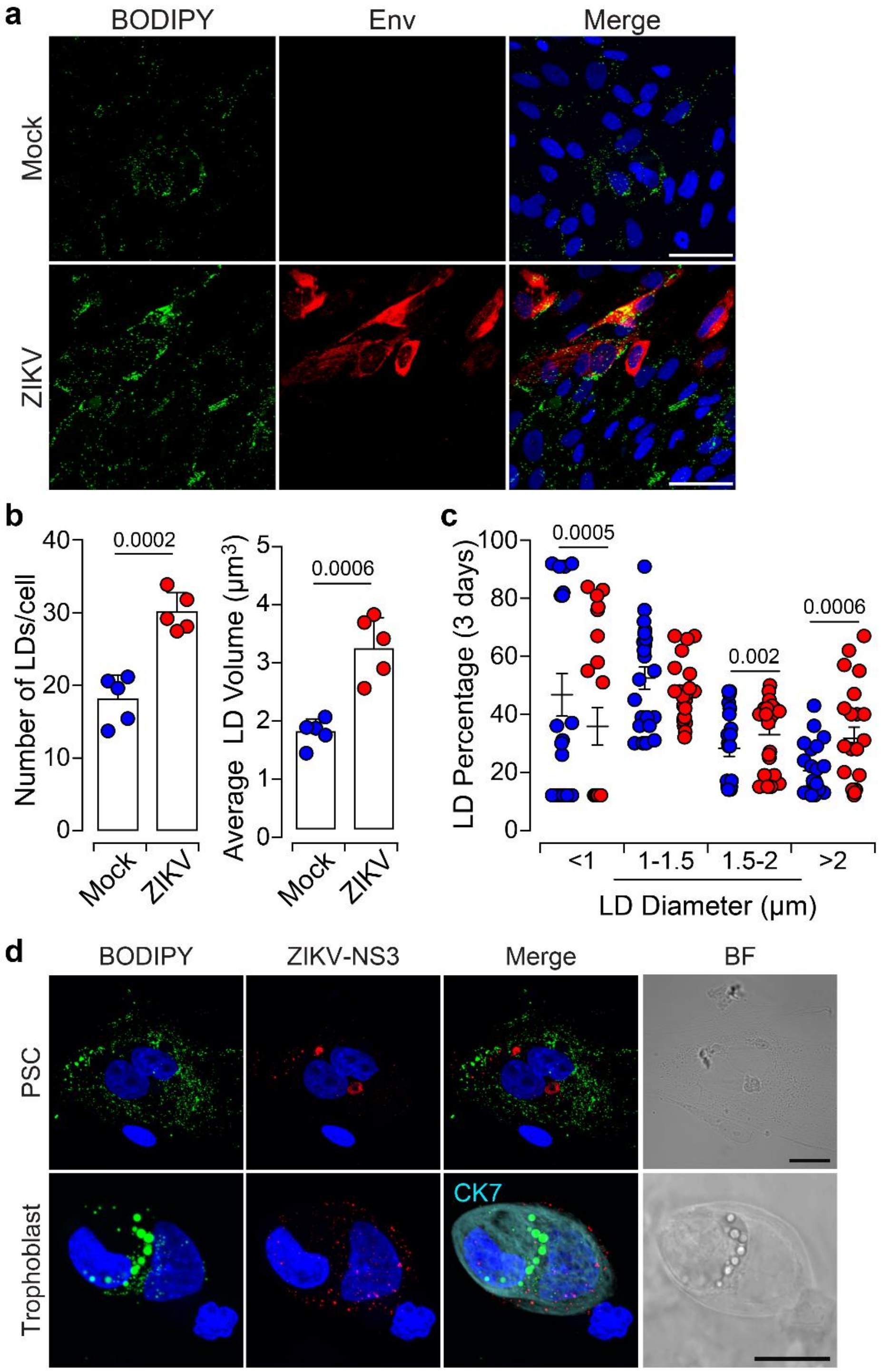

Supplementary Figure 4. ZIKV-Induced Alterations of LD Morphometric Features.

Freshly isolated primary placental cells infected with ZIKV at MOI of 1 for 3 days. **a** Maximum intensity projection of Z-stack images. LDs labeled with the BODIPY 493/503 dye (green), anti-flavivirus group antigen (Env, red), and DAPI stained nuclei (blue). Scale bar, 50μm. **b** Quantification of LD number per cell, average volume by Imaris software. LD morphometric features are analyzed in more than 400 cells for each placenta from 5 independent donors. Results are presented as mean values ± SEM. *P* values are computed using the two-tailed Student's t test. **c** Bar graph showing the percentage of LD size for each diameter range at the indicated time point following infection. Data are presented as mean values ± SEM. *P* values are computed using the two-tailed Student's t test. **d** Representative higher magnification images of placental stromal cells (PSC) and trophoblast cells. BODIPY 493/503 (green), anti-ZIKV NS3 protein (NS3, red) and anti-CK7 (cyan). Scale bar, 20μm. Data are representative of at least three independent experiments. Source data are provided as a Source Data file.

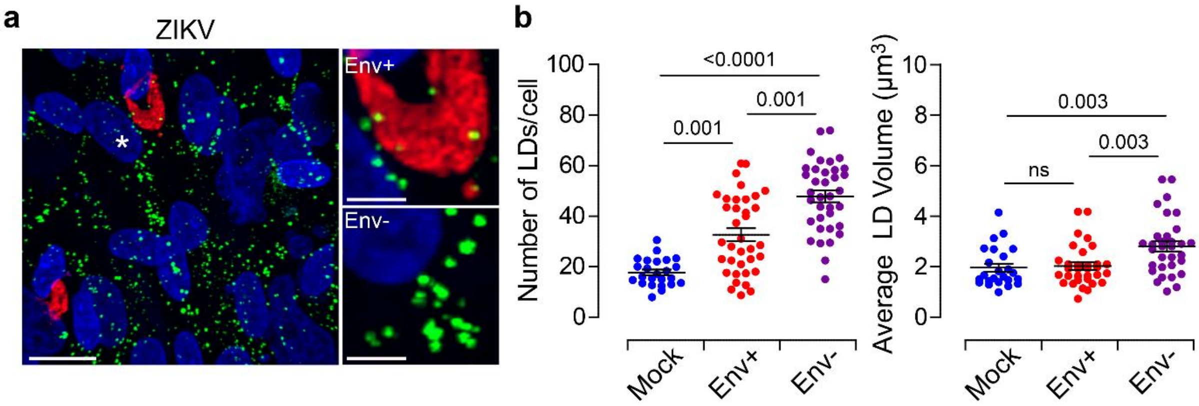

Supplementary Figure 5. Bystander Effect of ZIKV Infection on LD Biogenesis.

Placental cells were infected with ZIKV MOI of 1 for three days. **a** LD stained with the BODIPY 493/503 dye (green), anti-flavivirus group antigen staining (Env) distinguishes $ZIKV^{+}$ (red) from $ZIKV^{-}$ bystander neighboring cells (asterisk). High-magnitude images of LD are shown in the right panels. Scale bar, 20μm. High magnification image insets showing LDs in both $ZIKV^{+}$ (Env^{+}) and $ZIKV^{-}$ (Env^{-}) bystander, scale bar 5μm. **b** Morphometric measurements of LDs number and volume based on Z-stack projections of confocal images. Data from five different donors are shown as dot plot graphs as mean values ± SEM. Each data point represents the average number of LDs per cells and average volume from Mock- (blue) and $ZIKV^{+}$ cells (red) or $ZIKV^{-}$ bystander cells (violet). *P* values are computed using the one-way ANOVA with Dunnett's multiple comparison test. Source data are provided as a Source Data file.

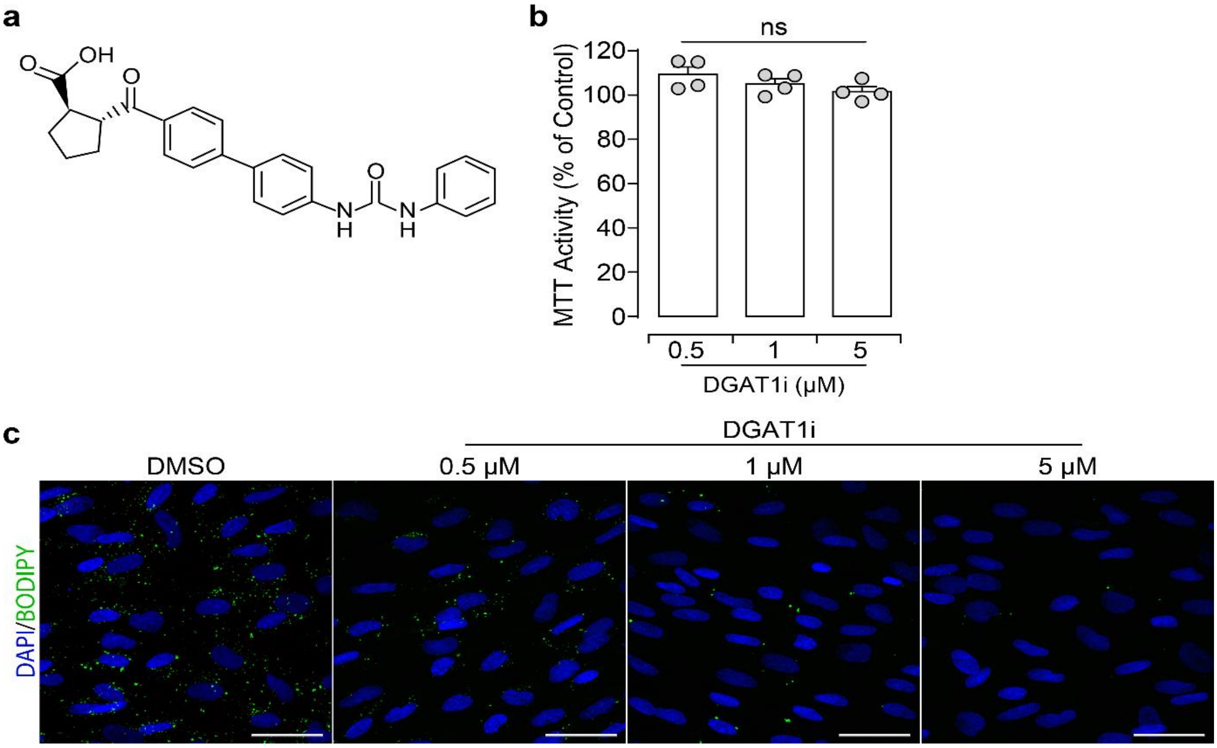

Supplementary Figure 6. Impact of DGAT1 inhibition on Placental Cells.

a Molecular structure of 2-[[4'-[[(phenylamino)carbonyl]amino][1,1'-biphenyl]-4-yl]carbonyl]-, (1R,2R)-, diacylglycerol acyltransferase 1 inhibitor (DGAT1i). **b** MTT cytotoxicity assay of primary placental cells treated with increasing amount of DGAT1i for 5 days. The same concentration of DMSO was added as a control. The cell viability was calculated as a percentage of DMSO control cells. All data are presented as mean values ± SEM of placentas from 4 independent donors. Significant difference between each group was calculated by one-way ANOVA, with Dunnett's multiple comparison test. No cytotoxicity of DGAT1i was observed at different concentrations. **c** Placental cells were treated with DGAT1i at different concentrations for 5 days. LDs were stained with the BODIPY 493/503 dye (green) and nuclei with DAPI (blue). Scale bar, 50µm. Source data are provided as a Source Data file.

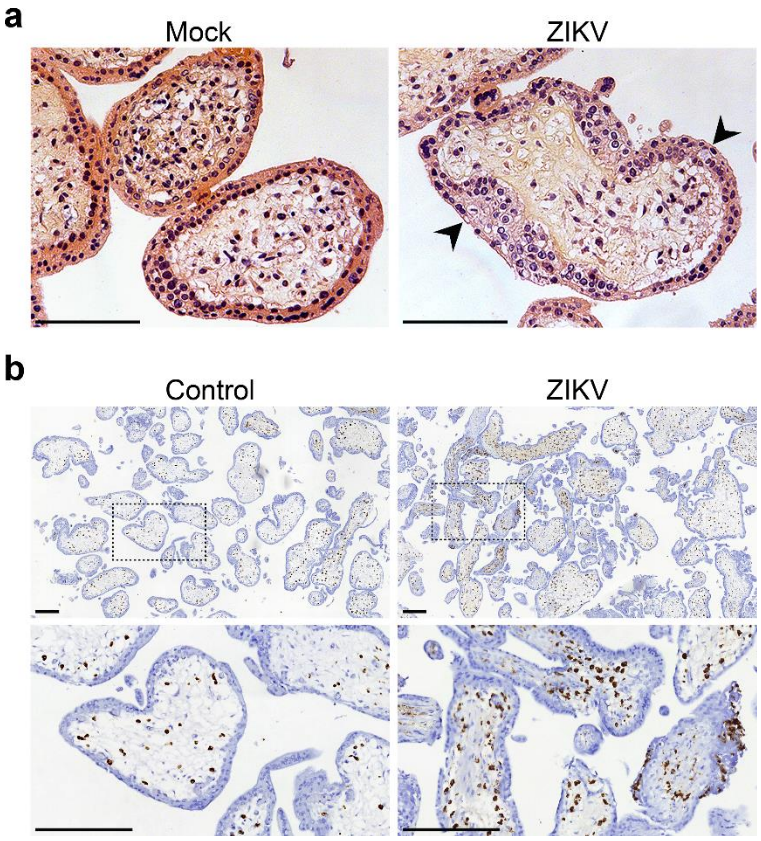

Supplementary Figure 7. ZIKV Induces Placenta Inflammation and Impacts Tissue Architecture.

a Histopathological analysis of HE-stained sections prepared from mock- and ZIKV-infected placenta explants 5dpi. Arrowhead points to alterations of tissue architecture with changes in trophoblast layers at the surface of the villi. Scale bar, 100µm. Data are representative of at least three independent experiments.

b IHC staining with anti-CD68 antibody (brown staining) in section from a congenital ZIKV-infected placenta and age-matched controls (10 weeks gestational age) from medical termination of pregnancy. High-magnification of the indicated areas are shown lower panels. Scale bar, 100µm.

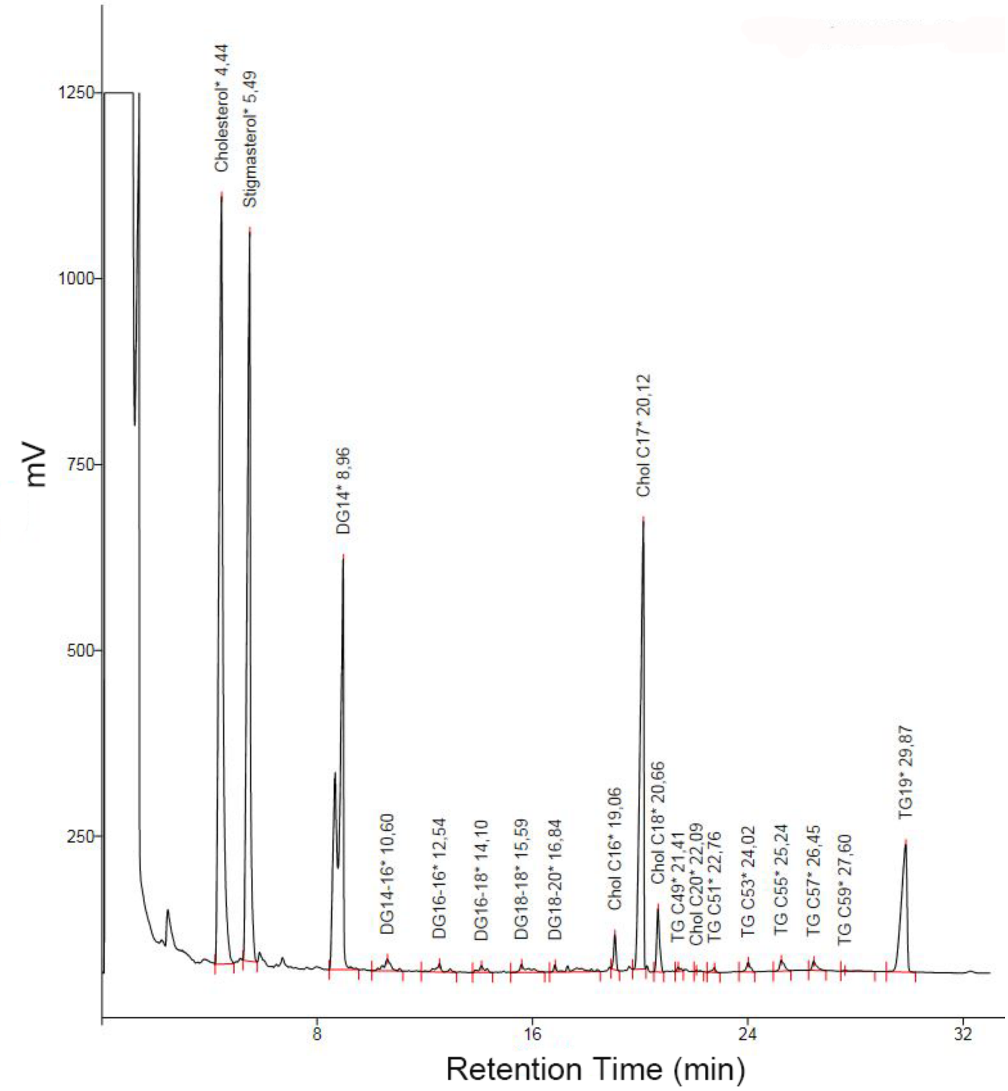

Supplementary Figure 8. Chromatogram of Neutral Lipid species from ZIKV Infected Placentas.

Diacylglycerol (DG), Triacylglycerol (TG), Cholesterol Ester (Chol). Retention time is indicated for each lipid specie.

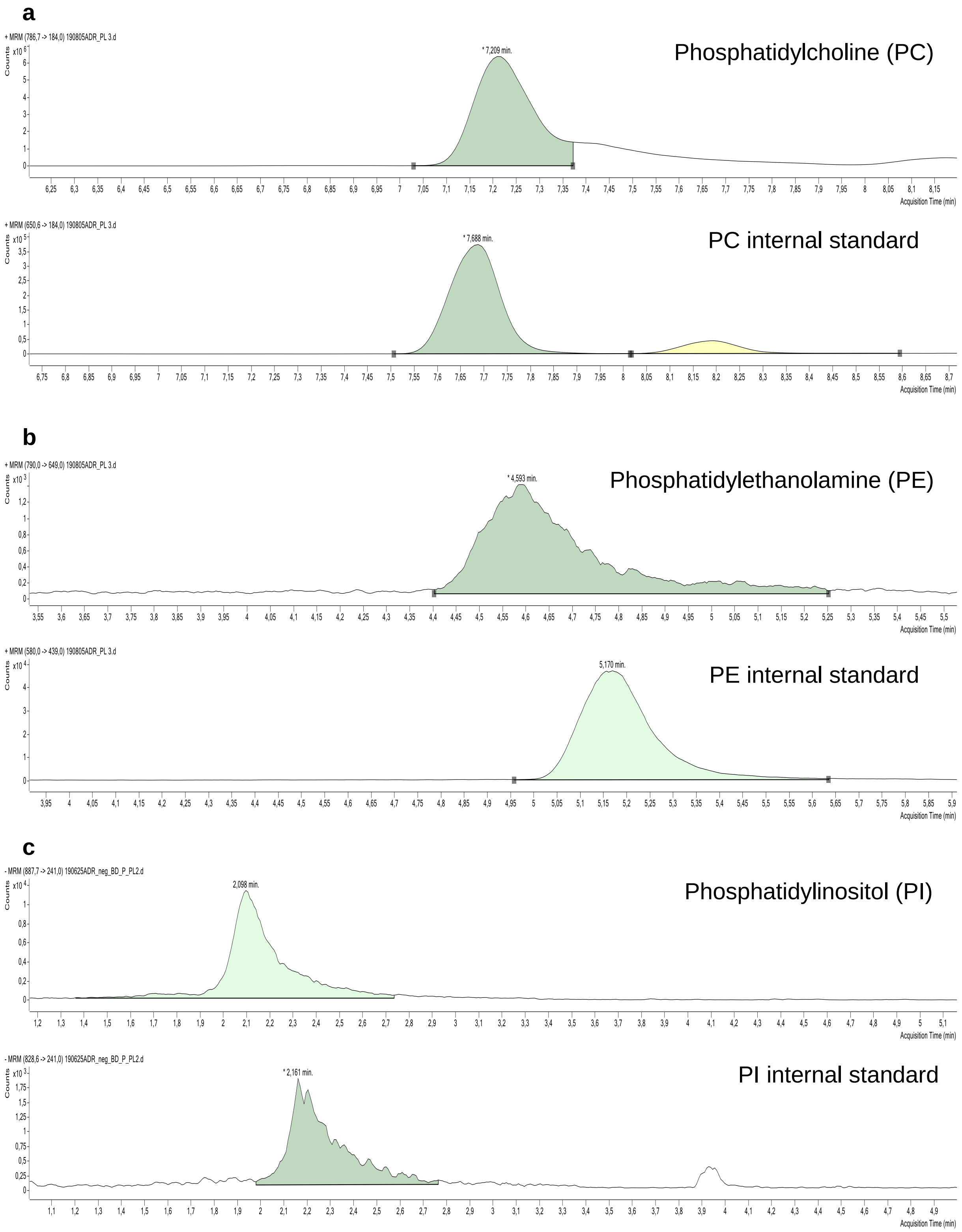

Supplementary Figure 9. Chromatogram of Phospholipids from ZIKV Infected Placentas.
Peaks of internal standards are given for each phospholipid. Acquisition time (min) is given for each peak.

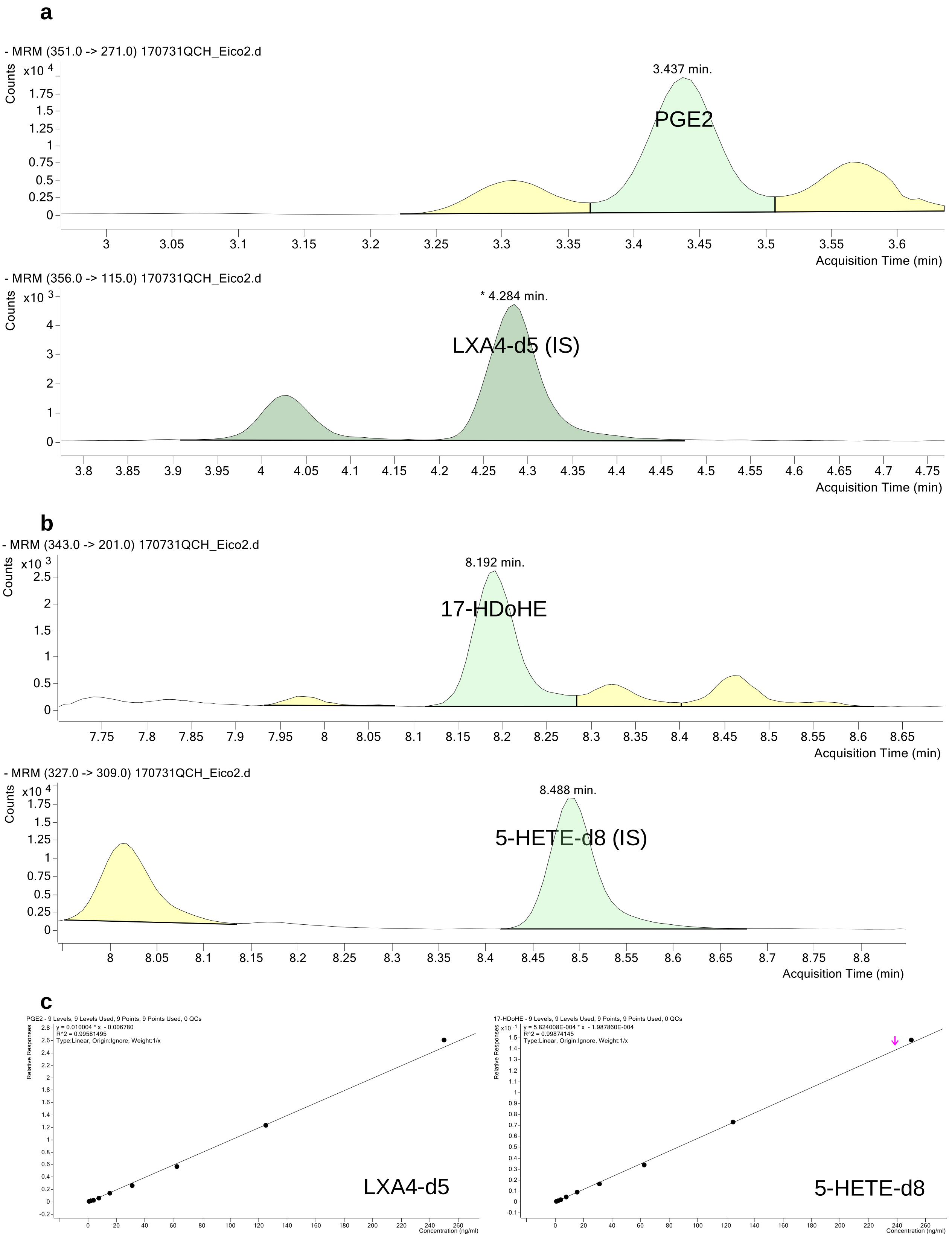

Supplementary Figure 10. Representative eicosanoid profiles from ZIKV-infected placentas.

(**a**) Prostaglandin E2 (PGE2) and (**b**) 17-Hydroxy-docosahexaenoic acid (17-HDoHE). Peaks of lipid mediators and their respective internal standards are shown. Acquisition times are indicated above peaks. (**c**) Calibration curves run with different concentration of the specific internal standard used for quantification of PGE2 and 17-HDoHE.

Supplementary Tables

Supplementary Table 1. qRT-PCR Primer Sequences

Target Gene	Primer Sequence (5’-3’)	
	Forward	Reverse
DGAT1	TCGCCTGCAGGATTCTTTAT	GCATCACCACACACCAGTTC
FAT/CD36	GCAGCAACATTCAAGTTAAGCAA	GGGTTACATTTTCCTTGGCTAGAA
FASN	CTTCCGAGATTCCATCCTACGC	TGGCAGTCAGGCTCACAAACG
HPRT	TGACACTGGCAAAACAATGCA	GGTCCTTTTCACCAGCAAGCT

Supplementary Table 2. Retention Time and Mass Value for Neutral Lipids

Target Lipid	Subspecies	Retention Time	Mass Value
DG	DG 14_16<br>DG 16_16<br>DG 16_18<br>DG 18_18<br>DG 18_20	10.60<br>12.54<br>14.10<br>15.59<br>16.84	540.9<br>569<br>597<br>625<br>653
TG	C49-TG (14_16_16)<br>C51-TG (16_16_16)<br>C53-TG (16_16_18)<br>C55-TG (16_18_18)<br>C57-TG (18_18_18)<br>C59-TG (18_18_20)	21.41<br>22.76<br>24.02<br>25.24<br>26.45<br>27.60	778<br>806<br>834<br>862<br>890<br>918
Cholesterol		4.44	386.64
Cholesterol Ester	CE-C16<br>CE-C18<br>CE-C20	19.06<br>20.66<br>22.09	625<br>649<br>673

Supplementary Table 3. Ion Precursor and Neutral Loss Scan for Phospholipids

Acquisition	Time Segment (min)	Phospholipid	Scan mode	m/z	Ion mode	Transition	CE (eV)	Neb (Psi)
1	0-3 min	Cer	Precursor ion	264	positive	500->700	25	25
	3-6 min	PE	Neutral loss	141	positive	570->870	20	25
	6-15 min	PC	Precursor ion	184	positive	640->915	30	25
	6-15 min	SM	Precursor ion	184	positive	555->820	25	25
2	0-20 min	PI	Precursor ion	241	negative	780->990	45	25
	0-20 min	PS	Neutral loss	87	negative	615->815	22	25

Mass Spectrometry (MS) conditions for phospholipid analysis.
Ion detected: $[M+H]^+$ and $[M-H]^-$. Delta EMV(+): 200.
Source Parameters: Spray Voltage: +4000 V. Source temperature: 325°C. Sheath gas temperature: 400°C. Nebulizer gas (Nitrogen) Flow: 10 L/min. Sheath gas (Nitrogen) Flow: 12 L/min.

Supplementary Table 4. MRM Transition for Phospholipid Analysis

Target Lipid	Phospholipids	MRM Transition
**PC**	*PC 13:0/13:0*	*650,7->184*
	PC 28:0	678,7->184
	PC 30:0	706,7->184
	PC 30:1	704,7->184
	PC 32:0	734,7->184
	PC 32:1	732,7->184
	PC 32:2	730,7->184
	PC 34:0	762,7->184
	PC 34:1	760,7->184
	PC 34:2	758,7->184
	PC 34:3	756,7->184
	PC 36:1	788,7->184
	PC 36:2	786,7->184
	PC 36:3	784,7->184
	PC 36:4	782,7->184
	PC 38:2	814,7->184
	PC 38:3	812,7->184
	PC 38:4	810,7->184
	PC 38:5	808,8 -> 184
	PC 38:6	806,7 -> 184
	PC 40:3	840,7->184
	PC 40:6	834,7 -> 184
**PE**	*PE 12:0/12:0*	*580,0 -> 439.0*
	PE 32:0	692,0 -> 551,0
	PE 32:1	690,0 -> 549,0
	PE 32:2	688,0 -> 547,0
	PE 34:0	718,0 -> 577,0
	PE 34:1	716,0 -> 575,0
	PE 36:1	746,0 ->605,0
	PE 36:2	744,0 ->603,0
	PE 36:3	742,0 -> 601,0
	PE 36:4	740,0 -> 599,0
	PE 38:2	772,0 -> 631,0
	PE 38:3	770,0 -> 629,0
	PE 38:4	768,0 -> 627,0
	PE 38:5	764,0 -> 623,0
	PE 38:6	766,0 -> 625,0
	PE 40:3	798,0 -> 657,0
	PE 40:5	794,0 -> 653,0
	PE 40:6	792,0 -> 651,0
	PE 40:7	790,0 -> 649,0

Target Lipid	Phospholipids	MRM Transition
**PI**	*PI 15:0/18:1 d7*	*828.6->241*
	PI 32:1	807,7->241
	PI 34:2	835,7 -> 241
	PI 34:2	833,7->241
	PI 36:0	865,7->241
	PI 36:1	863,7->241
	PI 36:2	861,7->241
	PI 36:3	859,7->241
	PI 38:1	891,7->241
	PI 38:2	889,7->241
	PI 38:3	887,7->241
	PI 38:4	885,7->241
	PI 38:5	883,7->241
	PI 40:2	917,7->241
	PI 40:3	915,7->241
	PI 40:4	913,7->241
	PI 40:5	911.7 -> 241
	PI 40:6	909.7 -> 241
**PS**	*PS 12:0/12:0*	*622,4->535,4*
	PS 32:0	734,4->647,4
	PS 34:0	762,4->675,4
	PS 34:1	760,4->673,4
	PS 34:2	758,4->671,4
	PS 36:0	790,4->703,4
	PS 36:1	788,4->701,4
	PS 36:2	786,4->699,4
	PS 36:3	784,4 -> 697,4
	PS 38:2	814,4->727,4
	PS 38:3	812,4->725,4
	PS 38:4	810,4->723,4
	PS 38:6	806.4->719,4
	PS 40:5	836,4->749,4
	PS 40:6	834,4->747,4
	PS 42:6	862,4 ->775,4
**Cer**	*Cer 18:1/15:0*	*506->264*
	Cer 18:1/16:1	518->264
	Cer 18:1/16:0	520->264
	Cer 18:1/18:0	548->264
	Cer 18:1/18:1	546->264
	Cer 18:1/20:0	576->264
	Cer 18:1/22:0	604->264
	Cer 18:1/24:0	632->264
	Cer 18:1/24:1	630->264
	Cer 18:1/26:1	658->264
	Cer 18:1/26:0	660->264
**SM**	*SM 18:1/12:0*	*647,6->184*
	SM 18:1/14:0	675,6->184
	SM 18:1/16:1	701,6->184
	SM 18:1/16:0	703,6->184
	SM 18:1/18:1	729,6->184
	SM 18:1/18:0	731,6->184
	SM 18:1/20:1	757,6->184
	SM 18:1/20:0	759,6->184
	SM 18:1/22:1	785,6->184
	SM 18:1/22:0	787,6->184
	SM 18:1/24:1	813,6->184
	SM 18:1/24:0	815,6->184

Internal standards are shown in red

MS Conditions for MRM Analysis

MS1 Resolution:
Widest (PI, PS,Cer),
Wide (PE), Unit (PC, SM)
MS2 Resolution: Widest
Time Filtering: 0.07 min
Dwell time: 10 ms

Supplementary Table 5.

Time Segment HPLC Retention Time and SRM Conditions for individual Eicosanoid

Segment	dwell time (ms)	Start Time (min)	Analytes	RT (min)	Ion precursor (m/z)	Fragmentor (V)	CE (eV)	IS
1	60	0	6kPGF1a	1.77	369	110	14	LxA4-d5
			TxB2	2.75	369	110	8	LxA4-d5
			PGE3	2.82	349	100	6	LxA4-d5
			11B-PGF2a	2.86	353	130	20	LxA4-d5
			PGF2a	3.3	353	140	16	LxA4-d5
			PGE2	3.57	351	110	8	LxA4-d5
2	60	3.64	LxB4	3.85	351	115	6	LxA4-d5
			PGD2	3.88	351	100	12	LxA4-d5
			RvD2	4.12	375	120	12	LxA4-d5
			LxA4-d5	*4.45*	*356*	*100*	*6*	
			LxA4	4.48	351	100	8	LxA4-d5
			RvD1	4.63	375	100	4	LxA4-d5
3	50	5.2	8isoPGA2	5.5	333	100	2	LtB4-d4
			LTB5	5.71	333	130	8	LtB4-d4
			PGA1	5.81	335	90	2	LtB4-d4
			7MaR1	6.14	359	105	2	LtB4-d4
			LtB4-d4	*6.24*	*339*	*120*	*6*	
			PD1	6.25	359	110	6	LtB4-d4
			LTB4	6.26	335	115	4	LtB4-d4
4	80	6.75	5,6-DiHETE	7.22	335	115	6	LtB4-d4
			18-HEPE	7.38	317	115	2	LtB4-d4
			15dPGJ2	7.61	315	90	2	LtB4-d4
5	40	7.75	13-HODE	7.97	295	135	8	5-HETE-d8
			9-HODE	8	295	135	8	5-HETE-d8
			15-HETE	8.21	319	100	2	5-HETE-d8
			17-HDoHE	8.33	343	110	2	5-HETE-d8
			14-HDoHE	8.47	343	115	2	5-HETE-d8
			8-HETE	8.49	319	100	4	5-HETE-d8
			12-HETE	8.53	319	100	2	5-HETE-d8
			5-HETE-d8	*8.63*	*327*	*100*	*2*	
			5HETE	8.69	319	100	4	5-HETE-d8
6	75	8.9	14,15-EET	9.21	319	100	2	5-HETE-d8
			5-Oxo-ETE	9.35	317	120	10	5-HETE-d8
			11,12-EET	9.49	319	100	2	5-HETE-d8
			8,9-EET	9.56	319	100	2	5-HETE-d8
			5,6-EET	9.63	319	100	2	5-HETE-d8

Internal standards are shown in red

Mass Spectrometry (MS) conditions

Electrospray ionization (ESI): Negative ion mode. Ion detected: $[M-H]^-$. Delta EMV (-): 200.
Source Parameters: Spray Voltage: -3500 V, Source temperature: 325 °C, Sheath gas temperature: 400°C. Nebulizer gas (Nitrogen) Flow: 10 L/min. Sheath gas (Nitrogen) Flow: 12 L/min.
Analyse MRM mode:

- MS1 Resolution: Wide
- MS2 Resolution: Widest
- Time Filtering: 0.04 min

Supplementary Methods

Protein Quantification

Tissues were snap-frozen in liquid nitrogen and stored at -80°C. Frozen tissues were crushed with a FastPrep ®-24 Instrument (MP Biomedical) in 1 mL HBSS (Invitrogen). After 2 crush cycles (6.5 m/s, 30 s), 50 µL were withdrawn for protein quantification using Bio-Rad Protein assay.

Internal Standards

All the internal standards (IS) for neutral lipids were purchased from Sigma Aldrich. IS for phospholipids were purchased from Avanti Polar Lipids (Alabaster, LA, USA). Deuterated IS for bioactive lipids were purchased from Cayman Chemicals (Ann Arbor, MI, USA).

Neutral lipid profiling

The equivalent 10 mg of tissue were extracted according to Bligh and Dyer method in dichloromethane/methanol/water (2.5/2.5/2.1:v/v/v), in the presence of IS. The following IS combination was used: stigmasterol (4 µg), glyceride dimyristoleate (4 µg) cholesteryl heptadecanoate (4 µg) and glyceryl trinonadecanoate (12 µg). The organic phase was evaporated to dryness and neutral lipids were separated through a Solid Phase Extraction (SPE) cartridge. Briefly, SiOH cartridge (200 mg, Macherey Nagel) was equilibrated with 2 mL of dichloromethane. Each dried extract was dissolved in 20 µL of 10 % methanol in dichloromethane then loaded individual SPE cartridges. Neutral lipids were eluted with 2 mL of the same mixture. Finally, extracts were concentrated and dissolved in 20 µL of ethyl acetate. 1 µL of the lipid extract was analyzed by gas-liquid chromatography on a FOCUS Thermo Electron system using Zebron-1 Phenomenex fused silica capillary columns (5 m X 0,32 mm i.d, 0.50 m film thickness). The oven temperature was programmed from 200°C to 350°C at a rate of 5°C per min and the carrier gas was hydrogen (0.5 bar). The injector and detector were at 315°C and 345°C respectively.

Examples of peak profiles are illustrated in supplementary Figure 8. Retention time and mass value for neutral lipid are summarized in Supplementary Table 2.

Profiling the main classes of phospholipids and Ceramide-Sphingomyelin

Lipids corresponding to 5 mg of tissue were extracted in dichloromethane/methanol/acetic acid (2.5:2.5:2 v/v/v), in the presence of the following amounts of IS: Cer d18:1/15:0 (16 ng), PE 12:0/12:0 (180 ng), PC 13:0/13:0 (16 ng), SM d18:1/12:0 (16 ng), PI 16:0/17:0 (30 ng), PS 12:0/12:0 (156.25 ng). Samples were then centrifuged at 900xg for 3 min. The organic phase was dried under nitrogen, then dissolved in 50 µL of methanol. Samples were analyzed using Agilent 1290 UPLC system coupled to a G6460 triple quadrupole spectrometer (Agilent Technologies, Les Ulis, France). Data acquisition and analysis were performed with Mass Hunter Qualitative Software (vB.05.00, Agilent Technologies).

Kinetex HILIC column (Phenomenex, 50 x 4.6 mm, 2.6 µm) were used for LC separations. The column temperature was controlled at 40°C. The mobile phase A was acetonitrile and phase B was 10 mM ammonium formate pH 3.2. The gradient was as follows: from 10 % to 30 % B in 10 min; 10-12 min, 100 % B; and then back to 10 % of B at 13 min for 1 min re-equilibrium prior to the next injection. The flow rate of mobile phase was 0.3 mL/min and the injection volume was 5 µL. An electrospray ionization (ESI) was employed in positive (for Cer, PE, PC and SM analysis) and negative ion mode (for PI and PS analysis). Nitrogen was used as collision gas. Needle voltage was set at + 4000 V. Several scan modes were used. To obtain the mass of different species, we analyzed lipid extracts with a precursor ion scanning of 184 m/z, 241 m/z and 264 m/z for PC/SM, PI and Cer respectively; and a neutral loss scan of 141 and 87 for PE and PS respectively. The collision energy (CE) optimums for Cer, PE, PC, SM, PI, PS were 25 eV, 20 eV, 30 eV, 25 eV, 45 eV, and 22 eV respectively. For each lipid family, individual molecular species were scanned with suitable Selected Reaction Monitoring (SRM) detection mode. Two Multiple Reaction Monitoring (MRM) acquisitions are necessaries for the differences of each PLs species. Peak areas were measured using Mass Hunter software. Data were treated using QqQ Quantitative (vB.05.00) and Qualitative analysis software (vB.04.00).
MS conditions are summarized in Supplementary Table 3-4. Examples of peak profiles are illustrated in supplementary Figure 9.

Profiling of Bioactive lipids

Equivalent 20 mg of tissue was used to extract bioactive lipids. In brief, 300 µL of cold methanol and 5 µL of IS (Deuterium labeled compounds of LxA4-d5, LtB4-d4, 5-HETE-d8 at the concentration of 400 ng/mL) were added to each sample. After centrifugation at 900xg for 15 min at 4°C, supernatants were transferred into 2 mL 96 deep well plates and diluted with 2 mL H_2O. Samples were then submitted to SPE using OASIS HLB 96-well plate (30 mg Sorbent/well, Waters) pretreated with 1 mL methanol and equilibrated with 1 mL 10% methanol. After sample loading, extraction plate was washed with 1 mL of 10 % methanol. Columns were dried thereafter under aspiration. Lipids mediators were eluted with 1 mL of methanol. Prior to LC-MS/MS analysis, samples were evaporated under nitrogen gas and reconstituted in 10 µL of methanol. 5 µL of the extract was injected in LC-MS/MS. LC-MS/MS method for rapid and concomitant quantification of pro-inflammatory and anti-inflammatory polyunsaturated fatty acid metabolites as previously described[1]. Briefly, lipid mediators were separated on a ZorBAX SB-C18 column (2.1 mm, 50 mm, 1.8 µm) (Agilent Technologies) using Agilent 1290 Infinity HPLC system (Agilent Technologies)-coupled to an ESI-triple quadruple G6460 mass spectrometer (Agilent Technologies). Data were acquired in MRM mode with optimized conditions (ion optics and collision energy). In order to increase the sensibility, the mass method was segmented and the dwell times were optimized for each segment. Peak detection, integration and quantitative analysis were done using Mass

Hunter Quantitative analysis software (Agilent Technologies) based on calibration curves established with increasing concentrations of commercially available eicosanoid standards.

MS conditions were indicated in Supplementary Table 5. Examples of peak profiles and calibration curves are illustrated in Supplementary Figure 10.

Quantification of lipid species

Relative quantification of neutral lipid and phospholipid lipid species was achieved by comparison of the peak area of individual lipids relative to their class-specific internal standards. Relative values above 10 mV cut-off were considered positive in our analysis. Data from relative peak area ratios were then normalized by protein amount in each sample[2,3].

For bioactive lipid quantification, deuterated IS were chosen to fit different metabolite families. For each bioactive species, absolute quantification was analyzed based on the calibration curves. Calibration curves were calculated by the IS method using the area ratio between the analyte and the internal standard[1]. In brief, a linear regression (1/X weight factor) was applied for each IS analyte. The linearity and accuracy of the detection were determined (Supplementary Fig. 10c). Finally, the limit of detection (LOD) were defined. Absolute quantification data were normalized by per mg of protein for each placenta sample. A signal-to-noise ratio (s/n) over 10 was included in the analysis. Additional details on Machine settings and LOD for different compounds have been published elsewhere[1].

Supplementary References

1 Le Faouder, P. *et al.* LC-MS/MS method for rapid and concomitant quantification of pro-inflammatory and pro-resolving polyunsaturated fatty acid metabolites. *J Chromatogr B Analyt Technol Biomed Life Sci.* 932, 123-133, doi:10.1016/j.jchromb.2013.06.014 (2013).

2 Chiappini, F. *et al.* Metabolism dysregulation induces a specific lipid signature of nonalcoholic steatohepatitis in patients. *Sci Rep* **7**, 46658, doi:10.1038/srep46658 (2017).

3 Stuani, L. *et al.* Stable Isotope Labeling Highlights Enhanced Fatty Acid and Lipid Metabolism in Human Acute Myeloid Leukemia. *Int J Mol Sci* 19, doi:10.3390/ijms19113325 (2018).
