# Supplementary material for: Metabolic reprogramming by Zika virus provokes inflammation in human placenta
Source: Nat Commun. 2020 Jun 11;11:2967. doi: 10.1038/s41467-020-16754-z (PMC7290035; doi:10.1038/s41467-020-16754-z)
Supplement: Supplementary file 3 — Reporting Summary [file 41467_2020_16754_MOESM3_ESM.pdf]

natureresearch

Corresponding author(s): Nabila Jabrane-Ferrat_NCOMMS-19-27017A

Last updated by author(s): Jan 24, 2020

# Reporting Summary

Nature Research wishes to improve the reproducibility of the work that we publish. This form provides structure for consistency and transparency in reporting. For further information on Nature Research policies, see Authors & Referees and the Editorial Policy Checklist .

## Statistics

For all statistical analyses, confirm that the following items are present in the figure legend, table legend, main text, or Methods section.

| n/a | Confirmed | |
|---|---|---|
| ☐ | ☒ | The exact sample size (*n*) for each experimental group/condition, given as a discrete number and unit of measurement |
| ☐ | ☒ | A statement on whether measurements were taken from distinct samples or whether the same sample was measured repeatedly |
| ☐ | ☒ | The statistical test(s) used AND whether they are one- or two-sided<br>*Only common tests should be described solely by name; describe more complex techniques in the Methods section.* |
| ☐ | ☒ | A description of all covariates tested |
| ☐ | ☒ | A description of any assumptions or corrections, such as tests of normality and adjustment for multiple comparisons |
| ☐ | ☒ | A full description of the statistical parameters including central tendency (e.g. means) or other basic estimates (e.g. regression coefficient) AND variation (e.g. standard deviation) or associated estimates of uncertainty (e.g. confidence intervals) |
| ☐ | ☒ | For null hypothesis testing, the test statistic (e.g. *F*, *t*, *r*) with confidence intervals, effect sizes, degrees of freedom and *P* value noted<br>*Give P values as exact values whenever suitable.* |
| ☒ | ☐ | For Bayesian analysis, information on the choice of priors and Markov chain Monte Carlo settings |
| ☒ | ☐ | For hierarchical and complex designs, identification of the appropriate level for tests and full reporting of outcomes |
| ☒ | ☐ | Estimates of effect sizes (e.g. Cohen's *d*, Pearson's *r*), indicating how they were calculated |

*Our web collection on statistics for biologists contains articles on many of the points above.*

## Software and code

Policy information about availability of computer code

| | |
|---|---|
| Data collection | LightCycler 480 (Roche Diagnostics, France) for RNA expression, Panoramic 250 viewer (3DHISTECH, Hungary) for histology, Zeiss LSM 710 confocal microscopy and Hitachi-7650 transmission electron microscopy for image acquisition. Lipid samples were acquired by gas-liquid chromatography on a FOCUS Thermo Electron system, Agilent 1290 UPLC system coupled to a G6460 triple quadrupole spectrometer and Agilent 1290 Infinity HPLC system-coupled to an ESI-triple quadruple G6460 mass spectrometer. Seahorse XFp analyzer (Seahorse Bioscience) was used for mitochondrial stress test. |
| Data analysis | Statistical analyses were performed using GraphPad Prism 7 (GraphPad Software). Zen 2009 Light edition software ( Carl Zeiss, Germany) was used for confocal microscopy data analysis. Panoramic CaseViewer 2.3 software (3DHISTECH, Hungary), Image and fluorescence quantification was performed by ImageJ 1.5J (NIH) or Imaris 7.2.3 software (Bitplane AG, Switzerland). Lipid data were analyzed using QqQ Quantitative vB.05.00 and Qualitative analysis vB.04.00 softwareor Mass Hunter Quantitative analysis vB.05.00 software (Agilent). Seahorse data analysis performed by Wave 2.6.0 software (Agilent). R-studio was used for heatmap analysis (R Studio, Inc). All figures were assembled using Adobe photoshop and Illustrator (Adobe 2019). |

For manuscripts utilizing custom algorithms or software that are central to the research but not yet described in published literature, software must be made available to editors/reviewers. We strongly encourage code deposition in a community repository (e.g. GitHub). See the Nature Research guidelines for submitting code & software for further information.

## Data

Policy information about availability of data

All manuscripts must include a data availability statement. This statement should provide the following information, where applicable:

- Accession codes, unique identifiers, or web links for publicly available datasets
- A list of figures that have associated raw data
- A description of any restrictions on data availability

All the data supporting the findings of this study are available within the article and its supplementary information files. Additional information are available upon request to the lead author.

# Field-specific reporting

Please select the one below that is the best fit for your research. If you are not sure, read the appropriate sections before making your selection.

☒ Life sciences ☐ Behavioural & social sciences ☐ Ecological, evolutionary & environmental sciences

For a reference copy of the document with all sections, see nature.com/documents/nr-reporting-summary-flat.pdf

# Life sciences study design

All studies must disclose on these points even when the disclosure is negative.

| | |
|---|---|
| Sample size | All experiments were conducted on age-matched placenta samples (7-11 weeks pregnancy) obtained from elective pregnancy termination (Approved by the French South-West & Outmer II ethical committee and registered at the French Ministry of Higher Education and Research under the number DC-2016-2772). Due to inter-donor variabilities at least 3-6 independent donors were used for each experiment. Samples sizes were selected based on previous experience to obtain statistical significance and reproducibility (Siewiera et al. 2013, 2015 and Gouilly et al. 2018). |
| Data exclusions | No exclusion criteria was applied. |
| Replication | The same protocoles were applied to process all the human samples. All the samples were preserved as decribed in the material and method section and were kept under the same conditions. Experimental findings were reliably reproduced in at least three independent experiments using clinical material from a minimum of three different placentas as indicated throughout the manuscript. |
| Randomization | All tissue explants in our experiments were processed and randomly distributed in the different study groups. |
| Blinding | Investigations for qRT-PCR, lipidome and histological analyses were blindly conducted. The corresponding groupe for each sample was attributed only during the final analysis.<br>The principal investigators were not blinded to the sample allocation or to the outcome assessment in experiments dealing with histology, immunofluorescence and electron microscopy. The data presented did not require the use of blinding. |

# Reporting for specific materials, systems and methods

We require information from authors about some types of materials, experimental systems and methods used in many studies. Here, indicate whether each material, system or method listed is relevant to your study. If you are not sure if a list item applies to your research, read the appropriate section before selecting a response.

## Materials & experimental systems

| n/a | Involved in the study | |
|---|---|---|
| ☐ | ☒ | Antibodies |
| ☐ | ☒ | Eukaryotic cell lines |
| ☒ | ☐ | Palaeontology |
| ☒ | ☐ | Animals and other organisms |
| ☐ | ☒ | Human research participants |
| ☒ | ☐ | Clinical data |

## Methods

| n/a | Involved in the study | |
|---|---|---|
| ☒ | ☐ | ChIP-seq |
| ☒ | ☐ | Flow cytometry |
| ☒ | ☐ | MRI-based neuroimaging |

## Antibodies

| | |
|---|---|
| Antibodies used | Anti-Flavivirus group antigen antibodies (D1-4G2-4-15, Merck-Millipore,MAB10216), polyclonal antibody anti-ZIKV NS3 protein (polyclonal, GenTex,Cat # GTX133309), trophoblast marker Cytokeratin 7 (CK7, Dako,Cat # M7018), mouse anti-human CD68 (Dako Cat # GA609). Goat Anti-mouse IgG2a Alexa Fluor 555 (Invitrogen,Cat # A21137), Goat Anti-mouse IgG1 Alexa Fluor 633 (Invitrogen,Cat # A21126),donkey anti-Rabbit IgG Alexa Fluor 555 (InvitrogenCat # A31572). |
| Validation | All antibodies from commercial sources undergo validation using immunofluorescence, immunohisstochemistry, flow cytometry and western-blotting to ensure specificity and clarify the experimental procedure for research uses. |

## Eukaryotic cell lines

Policy information about cell lines

| | |
|---|---|
| Cell line source(s) | Vero cell line was purchased from the ATCC |
| Authentication | Vero ATCC ® CCL-81™ |
| Mycoplasma contamination | Cells were tested negative for mycoplasma |
| Commonly misidentified lines (See ICLAC register) | No commonly misidentified cell line was used. |

## Human research participants

Policy information about studies involving human research participants

| | |
|---|---|
| Population characteristics | First-trimester placentas (7-11 weeks of gestation) were obtained from healthy women undergoing elective termination of pregnancy. |
| Recruitment | Human samples were collected from healthy donors randomly who provided written informed consent. |
| Ethics oversight | Human sample collection by Dr Jabrane-Ferrat, Inserm UMR1043 - CNRS UMR 5282 of Toulouse France, was approved by the French South-West & Outmer II ethical committee and registered at the French Ministry of Higher Education and Research. Collection declaration number DC-2016-2772 (dossier 1-16-34). Tissue samples were obtained with written informed consent from all participants in accordance with the Declaration of Helsinki guidelines. |

Note that full information on the approval of the study protocol must also be provided in the manuscript.
